# Immune Transcriptome of Cells Infected with Enterovirus Strains Obtained from Cases of Type 1 Diabetes

**DOI:** 10.3390/microorganisms8071031

**Published:** 2020-07-12

**Authors:** Anello Marcello Poma, Angelo Genoni, Francesco Broccolo, Maria Denaro, Alberto Pugliese, Fulvio Basolo, Antonio Toniolo

**Affiliations:** 1 Department of Surgical, Medical, Molecular Pathology and Clinical Area, University of Pisa, 56126 Pisa, Italy; mariadenaro86@gmail.com (M.D.); fulvio.basolo@med.unipi.it (F.B.); 2 Medical Microbiology, Department of Biotechnology and Life Sciences, University of Insubria, 21100 Varese, Italy; angelopaolo.genoni@uninsubria.it (A.G.); antonio.toniolo@uninsubria.it (A.T.); 3 Medical Microbiology, Department of Medical Sciences, University Milano Bicocca, 20126 Milano, Italy; francesco.broccolo@unimib.it; 4 Diabetes Research Institute, University of Miami, Miami, FL 33136, USA; apuglies@med.miami.edu; 5 Global Virus Network, 21100 Varese, Italy

**Keywords:** diabetes, virus, pathogenesis, autoimmunity, persistent infection, interferon, cytokine, IL18, IL32, MCP1

## Abstract

Enterovirus (EV) infection of insulin-producing pancreatic beta cells is associated with type 1 diabetes (T1D), but little is known about the mechanisms that lead the virus to cause a persistent infection and, possibly, to induce beta cell autoimmunity. A cell line susceptible to most enterovirus types was infected with EV isolates from cases of T1D and, for comparison, with a replication-competent strain of coxsackievirus B3. The transcription of immune-related genes and secretion of cytokines was evaluated in infected vs. uninfected cells. Acutely infected cells showed the preserved transcription of type I interferon (IFN) pathways and the enhanced transcription/secretion of *IL6, IL8, LIF, MCP1*, and *TGFB1*. On the other hand, infection by defective EV strains obtained from diabetic subjects suppressed IFN pathways and the transcription of most cytokines, while enhancing the expression of *IL8, IL18, IL32*, and *MCP1*. IL18 and IL32 are known for their pathogenic role in autoimmune diabetes. Thus, the cytokine profile of AV3 cells infected by diabetes-derived EV strains closely matches that observed in patients at the early stages of T1D. The concordance of our results with clinically verified information reinforces the hypothesis that the immune changes observed in type 1 diabetic patients are due to a hardly noticeable virus infection.

## 1. Introduction

Type 1 diabetes (T1D) is characterized by an irreversible impairment of glucose homeostasis due to the loss of insulin-producing pancreatic beta cells. Though T1D is considered a disease of the young, currently it is diagnosed in all decades of life. The disease originates from a complex interaction of genetic, immunological, and environmental factors such as virus infection of beta cells in the islets of Langerhans. Numerous studies have associated enterovirus (EV) infection with T1D, especially regarding the direct effect of the virus on pancreatic islets and its possible role in triggering islet-specific autoimmunity.

EV antigens have been detected in pancreatic islets of post-mortem T1D cases [1] and in pancreatic biopsies obtained from live patients [2]. At the time of clinical onset, interferon (IFN)-stimulated genes are expressed in the islets and human leukocyte antigen (HLA) class-I antigens are hyperexpressed [3]; memory CD8 T lymphocytes are among the infiltrating cells. Serologic studies have shown that antibodies that neutralize group B coxsackieviruses are statistically associated with the inception of beta-cell autoimmunity that heralds diabetes [4]. In addition, studies of children at genetic risk for T1D show that different EV types are present in stools prior to the development of islet autoantibodies [5]. Taken together, the data suggest that EV infection may represent a pre-requisite for the development of T1D [5,6,7,8,9].

The results of collaborative studies within the network of Pancreatic Organ Donors with Diabetes (nPOD; Miami, FL, USA) have reinforced this notion showing that EV infection subsists in the lymphoid and pancreatic tissues of T1D patients independently of time from diagnosis [10].

Since the disease is, in part, immune-mediated, it is central to identify how the infecting viruses may trigger autoimmunity. A variety of mechanisms may operate [11]. In the early phase of infection, innate immunity is activated by the recognition of pathogen-associated molecular patterns. This leads to the secretion of type-I interferons (IFNs) and pro-inflammatory cytokines. IFNs function in an autocrine manner by self-inducing antiviral responses in the infected cell, as well as in a paracrine manner by signaling the infection and inducing a virus-resistant state in neighboring cells. The type I IFN response activates the Janus kinase (JAK)-signal transducer and activator of the transcription (STAT) pathway which results in the transcription of IFN-stimulated genes (ISGs) responsible for the antiviral state. In a later phase, virus replication is accompanied by more or less pronounced cell damage, possibly accompanied by the release of self-antigens. These are taken up by antigen-presenting cells that, after processing, deliver self-peptides to lymphocytes. Autoreactive T cell clones may arise, and autoreactive B cells may start producing autoantibodies.

Within the islets, macrophages and dendritic cells are engaged in an inflammatory process in which many components of the immune system play a role (insulitis). Within endocrine epithelial cells, HLA-I molecules present antigens to immune cells and play a role in antiviral defense [3]. Similarly, HLA-II molecules are also involved in antigen processing and presentation [12].

Regarding EV infection, innate immunity factors and cytokines are expressed in human cells challenged by coxsackievirus B3 (an enterovirus of the B species). Infection may be followed by the upregulation of viral RNA sensors, IFN-stimulated genes (*IP-10, ISG15, OAS1, OAS2, Mx2*), and different cytokines (interleukin-6 (*IL6*), *IL8*, monocyte chemoattractant protein-1 (*MCP1*)) [13]. 

Studies in humans have shown that T1D is associated with a chronic type of EV infection [14,15]. Chronic infection markedly differs from the acute infection brought about by replication-competent EV strains. In fact, acute EV infection rapidly shuts off protein synthesis and is followed by cell death [16]. In contrast, the EV strains associated with diabetes are virus variants endowed with little replicative ability [14,15] and with the capacity of egressing the cell through “autophagosome-mediated exit without lysis”, i.e., within membranous vesicles [16]. Accordingly, diabetes-derived EV strains are characterized by slow replication, the continuous release of particles without manifest cytopathology, and the incessant activation of immune-related genes [17].

To investigate the consequences of infection by EV strains obtained from T1D cases, we analyzed the transcriptome of a reporter epithelial cell line infected with virus isolates from T1D cases. Transcripts of immune-related genes were compared in infected vs. non-infected cells. Results indicate that infection by diabetes-derived EV strains influences multiple pathways. Under persistent infection, the presentation of autoantigens may initiate an autoimmune response [17,18].

## 2. Materials and Methods

Instrumentation, consumables, cell lines, culture media, chemicals, molecular biology reagents, antibodies, commercial kits, software and applications are listed in Supplementary Table S1.

### 2.1. Human Samples

For virus isolation, spleen samples from the nPOD biobank [19] were shipped in liquid nitrogen to the laboratory. The observational study was approved by the Ethics Committee of Ospedale di Circolo and Fondazione Macchi (Varese, Italy; #2016/17233) and was performed in accordance with the Declaration of Helsinki and local regulatory laws. Once thawed, appropriate aliquots of each sample were used for virus detection. The remaining amounts were suspended in fetal bovine serum plus 10% DMSO, frozen gradually at −1 °C/min and stored in liquid nitrogen for subsequent analysis.

### 2.2. Cells and Virus Strains

AV3 cells (containing HeLa marker chromosomes and derived via HeLa contamination) and the RD, 1.1B4, VC3, and HEK-293 cell lines were obtained from The European Collection of Authenticated Cell Cultures (ECACC) and cultured at 37 °C in air with 5% CO_2_ using Dulbecco’s Modified Eagle Medium (DMEM)/F12 medium supplemented with L-glutamine, heat-inactivated 10% fetal bovine serum (FBS), and penicillin/streptomycin. Cell cultures were checked monthly for mycoplasma contamination (MycoAlert Plus mycoplasma kit). The Nancy strain of coxsackievirus B3 (CBV3) was originally obtained from American Type Culture Collection (ATCC, Manassas, VA, USA), propagated in AV3 cells, titrated, and stored at −70 °C. CBV3 was used as a reference strain for transcriptome analysis since this virus is pancreotropic and diabetogenic in mice [20,21,22] and supported by serologic studies in humans [23] as well as by the investigation of some T1D cases [24]. Regarding transcriptome analysis, CBV3 was chosen since complete data have been published on the cellular and transcriptome consequences of CBV3 infection in human cells [25,26]. For acute infection, confluent cultures in T25 flasks were infected at a multiplicity of infection of 3 using 1 mL of virus diluted in cell culture medium and incubated on a rocking platform at 37 °C. After 1 h, cell monolayers were washed 2× with warm medium, and flasks refilled with 5 mL warm medium. Cultures were then incubated at 37 °C for 4 h before harvesting. The persistent infection was investigated in AV3 cells exposed to undiluted EV isolates obtained from different human cases and incubated for 72 h. Total RNA was extracted from cell monolayers.

### 2.3. Expression of EV Receptors in the AV3 Cell Line

For indirect immunofluorescence, cell monolayers in Millicell EZ 4-well glass slides (Merck, Darmstadt, Germany) were fixed in phosphate-buffered saline (PBS) containing 4% paraformaldehyde. The expression of EV receptors was investigated by indirect immunofluorescence in uninfected AV3 cells using the antibodies listed in Supplementary Table S1. Alexa Fluor 488-goat anti-mouse IgG and fluorescein isothiocyanate (FITC) goat anti-rabbit IgG were used as secondary antibodies (Thermo Fisher, Waltham, MA, USA). Images were taken with a Nikon E80i microscope.

### 2.4. Detection of Enteroviruses in nPOD Spleen Samples

A published procedure for isolating and detecting EV strains causing chronic infection was followed [15]. Briefly, nPOD spleen samples (homogenates or live cells) were co-cultured with a mix of EV-susceptible cell lines (AV3, RD, 1.1B4, VC3, HEK-293) in order to enrich for the virus (2–3 passages). RNA was extracted from supernatants of cultures in T25 flasks, then reverse-transcribed (RT) with Superscript III reverse transcriptase and VILO master mix (Thermo Fisher). Four different EV-specific end-point RT-PCR assays were performed on each sample to detect possible EVs producing slow infection [8]. The LabChip GX Touch 24 analyzer (Perkin Elmer, Waltham, MA, USA) based on capillary electrophoresis was used to detect amplicons. A PCR test was deemed positive when an amplicon peak of the expected size was observed in the electropherogram. PCR amplicons were sequenced by the Sanger method. For indirect immunofluorescence, cell monolayers were fixed in PBS containing 4% paraformaldehyde. Cells expressing EV antigens were detected by staining with two mouse monoclonal antibodies (9D5 and 6-E9/2) that recognize the viral VP1 capsid protein. Alexa Fluor 488-labeled anti-mouse IgG was used as secondary antibody (Thermo Fisher). VP1 staining was deemed positive if granular cytoplasmic fluorescence was detected in infected cell cultures.

### 2.5. Immune Transcriptome of Control Uninfected and Infected AV3 Cells

For gene expression analysis, total RNA was purified from two T25 flasks containing monolayers of uninfected and infected AV3 cells using the RNeasy Mini Kit (Qiagen, Hilden, Germany). RNA quality was assessed by spectrophotometry (Trinean, Gentbrugge, Belgium). A gene expression assay was carried out as previously reported [27] using the nCounter Human Immunology v. 2 Panel (nanoString Technologies, Seattle, WA, USA) that allows analysis of 580 immune-related genes. Duplicate samples have been used; experiments have been repeated three times.

### 2.6. Release of Cytokines by Uninfected and Infected AV3 Cell Cultures

Supernatants of AV3 cell monolayers in T-25 flasks were collected from uninfected and infected cultures, incubated for 72 h, centrifuged to eliminate cell debris (10,000× *g* for 5 min), and stored at −70 °C. Supernatants were then examined for levels of 22 human cytokines using Luminex xMAP Technology assays capable of measuring GM-GSF, IFN-alpha, IFN-gamma, IL1-beta, IL2, IL3, IL4, IL5, IL6, IL7, IL8, IL10, IL12, IL17, IL18, MIP1-alpha, MIP1-beta, CCL2/MCP1, CCL5/RANTES, MIG, TNF-alpha, TNF-beta (Myriad RBM, Austin, TX, USA).

### 2.7. Statistical Analysis

Statistical comparisons of different experimental groups were performed using an unpaired two-tailed Student’s t test or a Mann–Whitney test (Graphpad Prism). *p* < 0.05 was considered statistically significant.

The normalization of target counts was performed by background thresholding as previously reported [28]. Similarity patterns of gene expression profiles were assessed by principal component analysis (PCA) and unsupervised hierarchical clustering based on Euclidean distance. The Bayes moderated t-statistics test was used to compute differentially expressed genes (DEG) using a false discovery rate (FDR) of 0.05 as cutoff. Gene set analysis was performed by the procedures of gage R package. Software and applications are listed in Supplementary Table S1.

## 3. Results

### 3.1. Network of Pancreas Organ Donors with Diabetes (nPOD): Isolation of Defective Enterovirus Strains from Spleen Samples

Spleen tissue from the nPOD biobank was used as a source for isolating T1D-associated defective EV strains. As shown in Table 1, four non-diabetic donors were selected together with four diabetic donors. The median age of non-diabetic donors was 11.2 years, and that of donors with diabetes was 22.5 years. Non-diabetic donors had median serum C-peptide levels of 3.45 ng/mL vs. 0.04 ng/mL in organ donors with diabetes. Four EV isolates obtained from donors with diabetes were investigated. Sequences of the 5’ UTR region of the genome and detection by immunofluorescence of the VP1 capsid protein in infected cell cultures confirmed that the infectious agents belonged to the Enterovirus genus.

### 3.2. Enterovirus Receptors in the AV3 Cell Line

**Figure 1:**
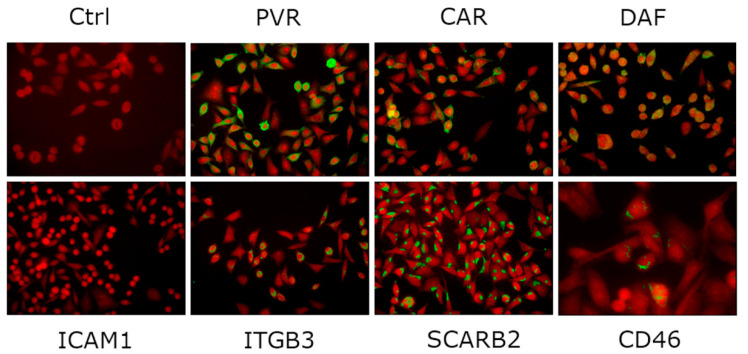
Immunofluorescent staining of AV3 cell monolayers with antibodies to representative enterovirus receptor molecules: poliovirus receptor (PVR), coxsackie-adenovirus receptor (CAR), complement regulator decay-accelerating factor (DAF), intercellular adhesion molecule 1 (ICAM1), integrin alpha-V/beta-3 (ITGB3); scavenger receptor class B member 2 (SCARB2); human herpesvirus-6 receptor CD46. Ctrl, cell monolayer incubated with secondary antibody only. Blue Evans counterstaining; original magnification, 250×.

The expression of EV receptor molecules in AV3 cells was analyzed by indirect immunofluorescence. AV3 cells express high levels of poliovirus receptor (PVR), coxsackie-adenovirus receptor (CAR), decay-accelerating factor (DAF), scavenger receptor class B member 2 (SCARB2), and integrin alpha-V/beta-3 (ITGB3) as well as low levels of P-selectin glycoprotein ligand-1 (PSGL-1) and integrin alpha-V/beta-6 (ITGB6, KREMEN1). In contrast, the major receptor for A-B rhinoviruses A and B, intercellular adhesion molecule-1 (ICAM-1), and the echovirus-1 receptor VLA-2 are not expressed [29]. Figure 1 shows immunofluorescent staining of five EV receptor molecules.

### 3.3. Infection of AV3 Cells with Replication-Competent CBV3 and with Defective EV Isolates from T1D Cases

AV3 cells were infected either with replication-competent CBV3 (CBV3) or with replication-defective EV strains from the spleen of donors with diabetes (Spl-EV). The latter strains were replicating very poorly and at minimal titers. However, though no manifest cytopathic effects were produced, the viral genome and viral antigens were consistently detected in cell cultures. There was no detection of 5’ terminal deletions in diabetes-derived EV strains but, as reported [15], scattered mutations and a few gaps were present in the 5’ non-translated portion of the genome. The uninfected controls consisted of uninfected cells (i.e., cells incubated with medium alone; CBV-3-Ctrl) or cells mock-infected with spleen preparations from donors without diabetes (Spl-Ctrl). The latter group did not contain a demonstrable virus (Table 1).

### 3.4. Gene Expression Analysis

Two experimental models were analyzed: (i) acute infection by CBV3, i.e., cells infected with a replication-competent CBV3 strain vs. uninfected control cells (CBV3 vs. CBV3-Ctrl); (ii) slow infection by EV isolates from T1D cases (Spl-EV) vs. mock-infected control cells (Spl-EV vs. Spl-Ctrl). Three independent experiments were performed. In each experiment, the CBV3 model was run in triplicate whereas the spleen-EV model was run in quadruplicate. Transcriptome results of the four experimental groups are available in Supplementary Table S2.

**Figure 2:**
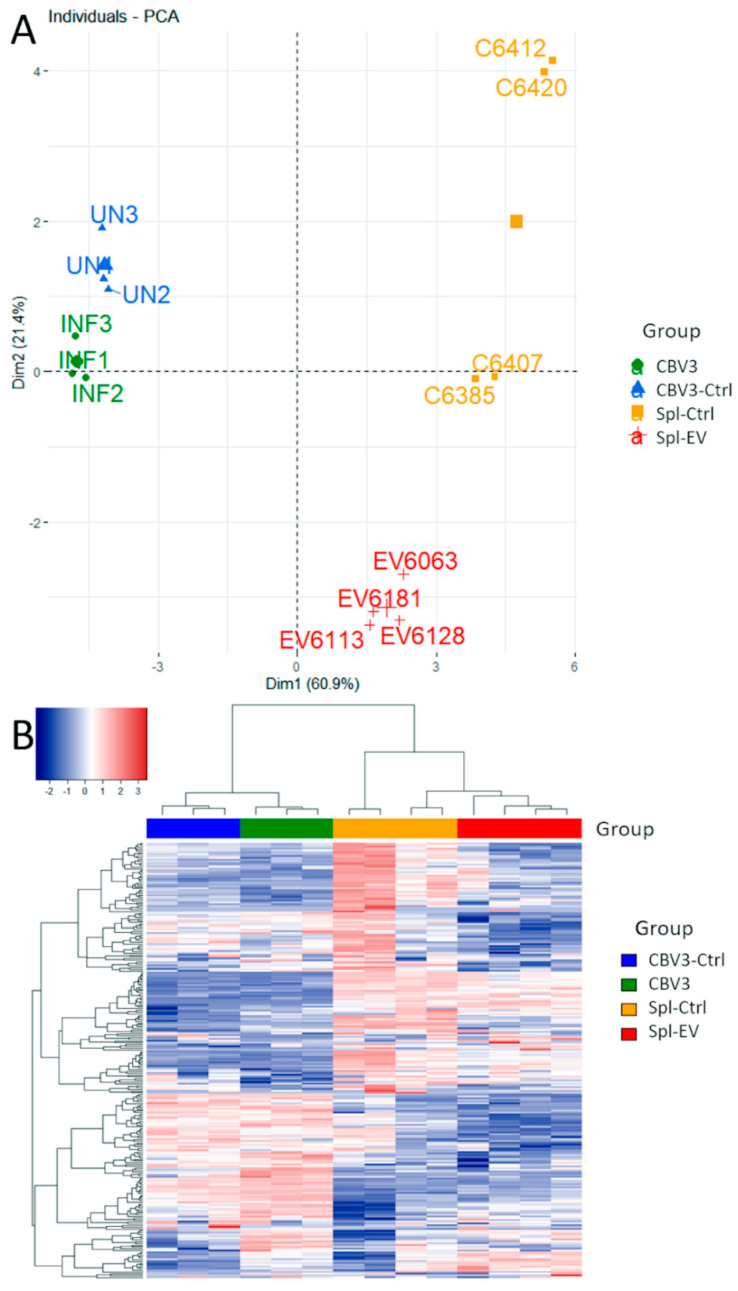
Principal component analysis (PCA) and unsupervised hierarchical clustering of gene expression data. (**A**) Principal components 1 and 2 accounted for more than 80% of variation and clearly separated the four groups; (**B**) In the heatmap, hierarchical clustering produced two main groups belonging to the acute infection model (left hand-side, 3 + 3 lanes) and the slow infection model (right hand-side, 4 + 4 lanes). Within each group, the expression profiles of uninfected (left lanes) and infected (right lanes) samples can be compared.

After normalization and thresholding, 243 genes were selected for further analyses. Principal component analysis (PCA) showed that the first two principal components accounted for over 80% of variation. These were used for plotting. Figure 2a shows that, for the two experimental groups, the variation in gene expression clustered into different quadrants. As shown in Figure 2b, unsupervised clustering of all samples produced two main clusters illustrated in the heatmap (Figure 2b). The first cluster (left-hand side, 3 + 3 lanes) refers to the model of acute infection with replication-competent CBV3; the second cluster (right-hand side, 4 + 4 lanes) refers to the model of slow infection with EV strains from diabetic subjects. Within each of the two clusters, the expression profiles distinguish the uninfected control samples (left) from the infected samples (right).

**Figure 3:**
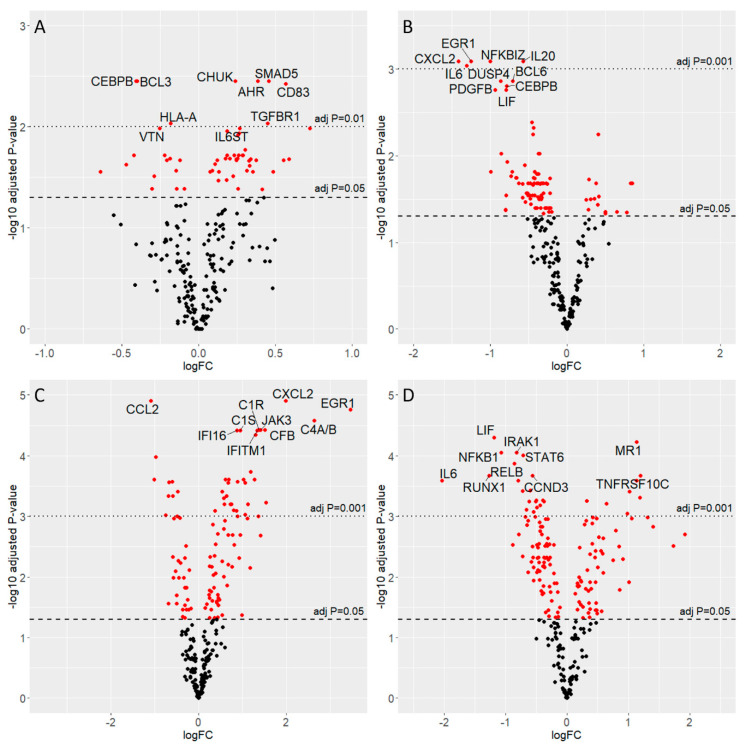
Volcano plots of four experimental groups. Volcano plots were obtained by plotting log2 fold change (x-axis) and -log10 of adjusted *p*-value (y-axis). In red are differentially expressed genes; dashed lines indicate the significance levels. For each comparison the top ten genes are displayed. Groups: (**A**) CBV3 vs. CBV3-Ctrl; (**B**) Spl-EV vs. Spl-Ctrl; (**C**) Spl-Ctrl vs. CBV3-Ctrl; (**D**) Spl-EV vs. CBV3.

In cells acutely infected with CBV3, 58 genes were differentially expressed (Figure 3a), with a predominance of upregulation (42 genes). Analysis of the involved pathways showed a trend for JAK-STAT activation. Interferon receptor genes (*IFNAR1, IFNAR2*) were upregulated together with cell adhesion molecules (*CD164, CD83*) and integrins (*ITGA6, ITGAE*), regulators of apoptosis and cell survival (*CASP2, CASP3, MCL1*), the *ETS1* transcription factor that controls the expression of cytokines and chemokines, and components of the proteasome (*PSMB5*) and of autophagic vesicles (*ATG5*). Notably, the expression of *SLC2A1* (a facilitative glucose transporter responsible for basal glucose uptake) was also enhanced in infected cells, possibly to sustain the energy levels adequate for virus production. Among cytokines, *IL6*, IL6 signal transducer (*IL6ST*), leukemia inhibitory factor (*LIF*), and transforming growth factor beta 1 (*TGFB1*) were upregulated. Transcription of the following genes was reduced: *BCL3* (transcriptional co-activator in association with NFKB), *CEBPB* (regulator of genes involved in immune and inflammatory responses), *KLRAP1* (activation of NK cells), *PTPN6* (regulator of innate immunity), and *IL1A* (signaling for immune and inflammatory responses).

In the slow infection (Spl-EV vs. Spl-Ctrl), 94 genes were differentially expressed (Figure 3b) with a dominance of downregulation (81 genes, i.e., 86.2%). *JAK1, STAT1* and *IFNAR1* were upregulated together with the adhesion molecule *CD164*, integrins *ITGA6* and *ITGAE, CASP3, ETS1*, *PSMB5*, and *SMAD5* (transcriptional modulator activated by Bone Morphogenetic Proteins, BMPs). Selected cytokines were also upregulated: the macrophage chemotactic factor *MCP1, IL18*, and *IL32*. Downregulation was seen for *TICAM1* (adapter for toll-like receptors to mediate NFKB and IRF activation and to induce apoptosis), *IKBKG* (NFKB activator that promotes inflammation, activation of IRF3, and TLR3- and IFIH1-mediated antiviral innate immunity), *TMEM173/STING1* (receptor that detects cytosolic nucleic acids and activates IFN-I), *SOCS3* (negative regulator of cytokine signaling), and *TAPBP* (transport of antigenic peptides for peptide loading on MHC class I molecules).

### 3.5. Interferon Pathways: Gene Transcription Changes in the Acute and the Slow Enterovirus Infection Models

Type I IFN pathways were compared in the acute and slow infection models (Table 2). The cytoplasmic RNA sensor IFIH1 plays a key role in the virus-induced pathway. The transcription of *IFIH1* was retained in both acute and slow infection. In acute infection, *TRAF3* (a regulator of NFKB and MAP kinases) and IKB kinase alpha (*IKBKA*) were enhanced. Downregulation was seen only for IKB kinase beta (*IKBKB*). In the paracrine IFN pathway, the IFN receptors *IFNAR1* and *IFNAR2* were conserved or potentiated in acute infection. *JAK1* and *STAT1* transcription was also potentiated, showing that the infected cells remain capable of binding type I IFN. In contrast, cells infected with defective EV strains showed reduced transcription of the receptor molecule *IFNAR1* and also of *JAK2, TYK2*, and *STAT2* [30]. Thus, chronically infected cells certainly have a flawed response to IFN.

### 3.6. Pathways Activated in the Acute and Slow Models of Infection

The gage package was used to analyze the expression patterns of overlapping genes and their associated pathways. In CBV3-infected cells, the JAK-STAT signaling pathway was activated. In cells infected by Spl-EV, four different pathways were activated: B-cell receptor signaling, T-cell receptor signaling, neurotrophin signaling, and regulation of actin cytoskeleton. The activated pathways are summarized in Table 3.

In cells exposed to the spleen-derived preparations (either containing or not containing the virus) the coagulation and complement cascades were activated. Upregulation of urokinase-type plasminogen activator (*PLAU*) was seen, concomitantly with the activation of complement factor I (*CFI*), *CF1, CFB, CFD, CF2, CF3, DAF, MCP*, and vitronectin (*VTN*). Gene expression changes were interpreted as follows: since in spleen-derived preparations the virus was present in minimal amounts, the preparations had to be employed at 1:10 dilution (*v/v*) thus exposing cells to minute debris. Nonetheless, the DEGs induced by these preparations (Figure 3c,d) were different and could be distinguished from those associated with infection by CBV3 or by diabetes-related EV strains.

### 3.7. Cytokine Transcription and Secretion in Acute and Slow Infection

In the acute infection model, *IL1B, IL6, LIF* (a cytokine of the IL6 family) and *TGFB1* were upregulated. In the slow infection model, the downregulation of cytokines was predominant (*GPI, IL1A* and *IL1B, IL6, LIF, MIF, IL20, CXCL2* and *TGFB1*). The transcription of the negative regulators of cytokines *SOCS3* and *CISH* (cytokine-inducible SH2-containing protein) was also reduced, possibly with a pro-viral role. Conversely, the transcription of *IL18, IL32* and *MCP1* was enhanced. The latter cytokines have a proinflammatory role, stimulate NK activity, increase the production of IFN-gamma, and are chemotactic for monocytes and basophils.

**Figure 4:**
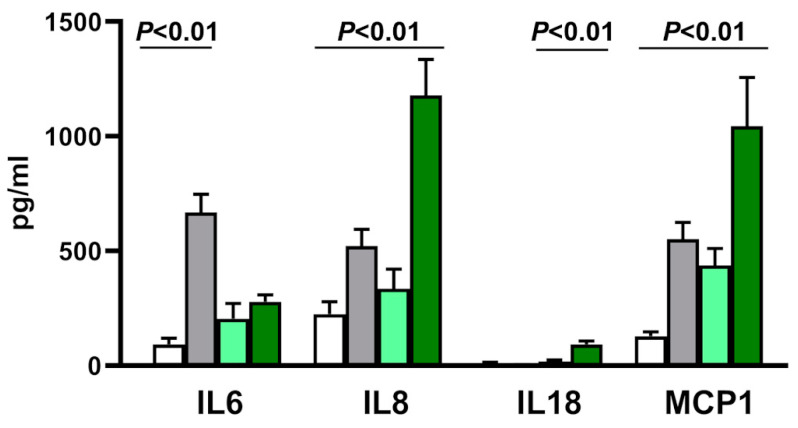
Release of IL6, IL8, IL18 and MCP1 by the reporter AV3 cell line incubated with medium alone (white bars), replication-competent CBV3 (gray), virus-free preparations from spleen of non-diabetic subjects (light green), and enterovirus strains obtained from the spleen of subjects with type 1 diabetes (dark green). The enhancement of IL18 release by cells infected with diabetes-related EV strains was minimal and is barely visible at the scale used in the graph. Experiments were done in quadruplicate. Error bars: mean with SD.

Data were confirmed, in part, by measuring the release of cytokines in infected vs. uninfected cells. Infection significantly influenced the release of only 4 out of the 22 measured cytokines: IL6, IL8, IL18, and MCP-1 (Figure 4). Compared to uninfected controls, cells infected with CBV3 produced higher levels of IL6, IL8, and MCP1. In contrast, cells infected with diabetes-related EV strains secreted enhanced levels of IL8, IL18, and MCP1, but not of IL6.

## 4. Discussion

A wide array of EV receptors are expressed in the AV3 cell line [29]. This line is, in fact, susceptible to many of the 115 human EV types recognized so far [31]. Infection by CBV3, a well-known member of the B species, has been investigated in detail in myocardial [25] and neural cells [26]. While replication-competent CBV3 reproduced at high titers in AV3 cells, the defective EV strains isolated from diabetes cases replicated at very low levels and did not induce manifest cytopathology. These viruses were, however, infectious since enteroviral proteins were expressed, the genome was replicated, and infectivity was neutralized by specific antibodies [15].

The study aimed to check whether persistent EV infection could change the expression of immune genes in ways capable of promoting inflammation and the response to self-antigens. Comparing acute CBV3 infection to the slow infection caused by diabetes-related EV strains revealed interesting differences.

Upon the recognition of viral pathogens by RNA sensors that include MDA-5 and TLR3, type I IFNs are produced. The binding of enteroviral RNA by MDA5 results in its association with mitochondrial antiviral signaling adaptor protein (MAVS) that binds to the TNF receptor-associated factor 3 (TRAF3). TRAF3 recruits and activates TANK-binding kinase 1 (TBK1) and inducible IKB kinase (IKK), leading to the phosphorylation of IFN regulatory factor 3 (IRF3) resulting in its translocation to the nucleus and transcription of IFN genes.

In acutely infected cells, the IFN induction pathway was preserved. Components of the IFN signaling pathway (i.e. the ability to respond to extracellular IFN) were not only preserved at the transcription level, but potentiated. Notably, the transcription of *JAK1* and *STAT1* was enhanced both in the acute and the slow infection models. Inversely, in cells infected by defective EV strains nearly all components of the IFN induction pathway were downregulated, including *IRF3*. In the paracrine IFN signaling pathway, the transcription of *IFNAR1, JAK2, TYK2* and *STAT2* was reduced. Thus, infection by diabetes-related EV strains harmed both the IFN-inducing and the IFN-responsive pathways, furthering viral persistence [30,32].

In agreement with our observations, previous studies showed that picornaviruses are equipped to interfere with the IFN-mediated antiviral state not only at the transcriptional level, but also at the protein level. The Theiler virus (a cardiovirus that causes chronic encephalomyelitis in mice) has been shown to interfere with transcription at two steps: (a) phosphorylation of IRF3 by TBK1 and IKKE, a process involving a complex with TRAF3; (b) nuclear entry downstream of IRF3 and binding to the IFN-beta gene promoter upstream of IRF3 [33]. In addition, at the protein level, enteroviral 3C protease binds MDA5, inhibiting its interaction with MAVS and blocking the IFN signaling pathway. The 3C protease also cleaves TAK1 (TGFB-activated kinase 1), resulting in the inhibition of NFKB activation, a host response critical for TLR-mediated signaling [34].

Previous studies of acute enteroviral infection showed that: (a) cultured neural cells infected with CBV3 had enhanced secretion of IL6, IL8, and MCP1 and the upregulation of SOCS3, a suppressor of cytokine signaling [26]; (b) human pancreatic islets infected with CVB3 and CVB4 secreted high amounts of IL6, IL8, MIP-alpha and -beta, and TNF; (c) human pancreatic beta cells infected with CBV4 showed the activation of innate immune pathways including the expression of IFN (alpha, beta, lambda), IL6, IL8, and IL27 and also showed a defective insulin response to glucose [35]; (d) patients with hand-foot-and-mouth disease caused by EV-A71 and EV-A6 had significantly increased serum levels of IL5, IL7, IL13, IL16, IL18, IP10, NGF-beta, PDGF-beta, and TGFB1 [36].

Our results on acute CBV3 infection are in line with the observations summarized above. In fact, acutely infected cells showed enhanced transcription and/or secretion of *IL6, IL8, LIF, MCP1*, and *TGFB1*. On the other hand, infection by diabetes-related EV strains reduced the expression of most cytokines including IFNs and *IL6*, but enhanced the expression of *IL8, IL18, IL32*, and *MCP1*. Thus, the expression of *IL18* and *IL32* may distinguish slow infection by diabetes-related EV strains from the acute model of infection.

Interestingly, pathogenetic studies of T1D—a disease in which persistent enteroviral infections are suspected—have provided results compatible with those observed for slow infection due to diabetes-related EV strains. Diabetes studies have indicated that: (a) IL6 is normally expressed in islet beta and alpha cells, but its production is reduced in the pancreas of diabetics [37]; (b) elevated levels of IL-18 are found in the blood cells of T1D patients and may promote NK cell activation and abrogation of the suppressive capacity of T regulatory cells [38]; and (c) peripheral blood mononuclear cells of children who developed beta-cell autoimmunity have upregulated *IL32* transcripts before the appearance of T1D-associated autoantibodies [39]. Finally, increased expression of MCP1 has been reported in newly diagnosed T1D children [40]. MCP-1 orchestrates the migration of myeloid and lymphoid cells during physiological immune defense as well as in autoimmune diseases [41].

In closing, the cytokine profile of AV3 cells infected by diabetes-related EV strains closely matches what is observed in patients at the early stages of T1D. Hence, our results reinforce the rationale that a long-standing viral infection of beta cells (perhaps in a slowly replicating form) may be one of the mechanisms leading to progressive beta cell loss in type 1 diabetes.

Our study has two major limitations: (a) a transformed epithelial cell line not adequately mimicking pancreatic beta cells was used as reporter, and (b) only a few diabetes-related EV strains have been examined so far. Large studies might clarify whether, upon persistent infection, the variation of immune gene expression is linked either to differences among the diabetes-derived isolates or to other factors. The nPOD biobank is an abundant source of multiple tissues for studying diabetes cases in comparison to non-diabetic controls. With these limitations in mind, transcriptome analysis appears a valuable tool for analyzing the consequences of viral infection in diseases of unknown etiology.

## Figures and Tables

**Table 1:** Investigated nPOD cases. Clinical characteristics of virus-free non-diabetic cases and diabetic cases from which enterovirus strains were obtained.

nPOD(miao) Case No.	Gender	Age (miao) (Years)	Clinical Diagnosis	Diabetes Duration (Years)	C-Peptide (ng/mL)	Virus
*Enterovirus-negative*						
6385	M	10.9	no diabetes	NA ^3^	1.55	no
6407	F	4.6	no diabetes	NA ^3^	5.35	no
6412	F	17.6	no diabetes	NA ^3^	5.60	no
6420	M	11.5	no diabetes	NA ^3^	1.27	no
*Enterovirus-positive*						
6063	M	4.4	T1D ^1^	3.0	0.04	Enterovirus
6113	F	13.1	T1D ^1^	1.6	0.04	Enterovirus
6128	F	33.8	T1D ^1^	31.5	0.04	Enterovirus
6181	M	31.9	AAb-pos ^2^	NA ^3^	0.06	Enterovirus

^1^ T1D, type 1 diabetes; ^2^ AAb-pos, positive for one or more diabetes-related autoantibodies (GAD, INS, IA2, ZnT8); ^3^ NA, not applicable.

**Table 2:** Type I interferon pathways. Gene transcription changes in the acute and the slow enterovirus infection models.

Gene	Gene Function	CBV3 vs. CBV3-Ctrl	Spl-EV vs. Spl-Ctrl
		Up or Down Regulation	Up or Down Regulation
***Type I IFN Induction Pathway (Response to Viral RNA)***			
*IFIH1 (MDA5)*	Cytoplasmic sensor of viral nucleic acids. Major role in sensing viral infection and in the activating the cascade of antiviral responses including the induction of type I IFNs and proinflammatory cytokines.	-	-
*TLR3*	Key component of innate and adaptive immunity, a nucleotide-sensing TLR activated by dsRNA. Acts via the adapter TICAM1.	-	-
*TICAM1*	Component of a multi-helicase-TICAM1 complex acting as a cytoplasmic sensor of viral dsRNA. Activates a cascade of antiviral responses, including proinflammatory cytokines.	-	↓
*TBK1*	Following the activation of toll-like receptors by viral or bacterial components, associates with TRAF3 and TANK and phosphorylates the IFN regulatory factors IRF3 and IRF7 and DDX3X. This activity allows the subsequent nuclear translocation of IRFs leading to the transcriptional activation of type I IFNs and pro-inflammatory cytokines. Activates IRF3 by phosphorylating innate adapters MAVS, TMEM173/STING, TICAM1 thus leading to the recruitment of IRF3.	-	-
*IFI16*	After binding to viral DNA in the cytoplasm, recruits TMEM173/STING and mediates the induction of IFN-beta. Has anti-inflammatory activity and inhibits the activation of the AIM2 inflammasome.	-	↓
*TMEM173 (STING1)*	Facilitator of innate immune signaling that acts as a sensor of cytosolic DNA from viruses and bacteria and promotes the production of type I IFN.	-	↓
*IRAK1*	IL1 receptor-associated kinase 1; phosphorylates interferon regulatory factor 7 (IRF7) to induce its activation and translocation to the nucleus, resulting in the transcriptional activation of type I IFN genes.	-	↓
*TRAF1*	Adapter molecule that regulates the activation of NFKB and JNK.	-	-
*TRAF2*	Regulates the activation of NFKB and JNK. Regulates cell survival and apoptosis.	-	↓
*TRAF3*	Regulates pathways leading to the activation of NFKB and MAP kinases. Regulates B-cell survival. Part of the signaling pathways leading to the production of IFN and cytokines. Role in T-cell dependent on immune responses.	↑	-
*TRAF4*	Activation of NFKB and JNK in response to signaling through TLRs. Regulates cell survival and apoptosis.	-	↓
*TRAF5*	Mediates the activation of NFKB.	-	↓
*TRAF6*	Activation of NFKB and JUN. Role in dendritic cell maturation and/or activation.	-	↓
*IKBKA (IKKA)*	Ikappa kinase (IKK) is an enzyme complex that is part of the NFKB signaling pathway. The IKK complex is comprised of three subunits: alpha, beta, and gamma. The alpha and beta subunits are catalytically active whereas the gamma subunit has regulatory functions.	↑	-
*IKBKB (IKKB)*		↓	↓
*IKBKG (NEMO)*		-	↓
*IKBKE (IKKE)*	Noncanonical IKB kinase (IKK) that is essential for regulating antiviral signaling pathways.	-	-
*NFKB1*	NFKB is a homo- or heterodimeric complex formed by the Rel-like domain-containing proteins RELA/p65, RELB, NFKB1/p105, NFKB1/p50, REL, and NFKB2/p52 and the heterodimeric p65-p50 complex. Dimers bind at kappa-B sites in the DNA of target genes and individual dimers have distinct preferences for different kappa-B sites.	↑	**↑**
*NFKB2*	NFKB2 has dual functions such as the cytoplasmic retention of attached NFKB proteins by p100 and the generation of p52 by a cotranslational processing.	-	↓
*NFKBIA*	Member of the NFKB inhibitor family. The protein interacts with REL dimers to inhibit NFKB/REL complexes which are involved in inflammatory responses.	-	↓
*NFKBIZ*	Member of the ankyrin-repeat family of proteins known to play a role in inflammatory responses. Activates IL6 but decreases TNF-alpha production.	-	↓
*RELA*	The NF-kappa-B heterodimeric RELA-NFKB1 and RELA-REL complexes function as transcriptional activators. The NFKB homodimeric RELA-RELA complex activates IL-8 expression.	**↑**	-
*RELB*	The NFKB heterodimeric RelB-p50 and RelB-p52 complexes are transcriptional activators. RELB is required for both T and B lymphocyte maturation and function.	-	-
*IRF3*	Key transcriptional regulator of type I IFN-dependent immune responses against viruses. Regulates the transcription of type I IFN genes and IFN-stimulated genes by binding to an IFN-stimulated response element (ISRE).	-	↓
*IFITM1*	IFN-induced antiviral protein which inhibits the entry of viruses to the cytoplasm, permitting endocytosis, but preventing subsequent viral fusion and the release of viral contents into the cytosol.	-	↓
*Type I IFN signaling pathway (response to IFN)*			
*IFNAR1*	Heterodimer with IFNAR2. Type I IFN binding activates the JAK-STAT signaling cascade and triggers the tyrosine phosphorylation of proteins including JAKs, TYK2, STAT, and the IFNR alpha- and beta-subunits themselves.	-	↓
*IFNAR2*	Associates with IFNAR1 to form the type I IFN receptor. Involved in IFN-mediated STAT1, STAT2, and STAT3 activation. Isoforms 1 and 2 are involved in signal transduction due to their association with JAK1.	**↑**	-
*JAK1*	Tyrosine kinase of the non-receptor type, involved in the IFN-alpha/beta/gamma signal pathway.	**↑**	**↑**
*JAK2*	Tyrosine kinase of the non-receptor type involved in different processes (cell growth, differentiation, histone modifications). Mediates signaling events in both innate and adaptive immunity. In the cytoplasm, mediates signal transduction via association with type II receptors (IFN-alpha, IFN-beta, IFN-gamma, and multiple interleukins).	-	↓
*TYK2*	Tyrosine kinase; associates with the cytoplasmic domain of type I and type II cytokine receptors and promulgates cytokine signals by phosphorylating receptor subunits. Component of type I and type III IFN signaling pathways.	-	↓
*STAT1*	Transcription activator that mediates cellular responses to IFNs, cytokines, and growth factors. Following type I IFN binding to cell receptors, signaling via protein kinases leads to the activation of TYK2 and JAK1 and to the tyrosine phosphorylation of STAT1 and STAT2. Phosphorylated STATs associate with ISGF3G/IRF-9 the ISGF3 complex transcription factor that enters the nucleus and promotes the transcription of IFN-stimulated genes which drive the cell in an antiviral state.	**↑**	**↑**
*STAT2*	Signal transducer and activator of transcription that mediates signaling by type I IFNs.	-	↓

**Table 3:** Significantly upregulated pathways in the acute infection and slow infection models.

Group Comparison	Pathway	Statistical Mean ^1^	q-Value
CBV3 vs. CBV3-Ctrl	hsa04630 JAK-STAT signaling pathway	1.33	0.2741
Spl-EV vs. Spl-Ctrl	hsa04662 B cell receptor signaling pathway	1.01	0.1844
	hsa04660 T cell receptor signaling pathway	0.96	0.1844
	hsa04810 Regulation of actin cytoskeleton	0.97	0.1844
	hsa04722 Neurotrophin signaling pathway	0.95	0.1844
Spl-Ctrl vs. CBV3-Ctrl	hsa04610 Complement and coagulation cascades	2.68	<0.0001
Spl-EV vs. CBV3	hsa04610 Complement and coagulation cascades	2.65	<0.0001

^1^ Quantitative estimate of pathway deregulation.

## Supplementary Materials

The following are available online at https://www.mdpi.com/2076-2607/8/7/1031/s1, **Table S1**. List of consumables, chemicals, cell lines, viruses, culture media, molecular biology reagents, antibodies, commercial kits, instruments, applications, and software, **Table S2**. Differential gene expression results.
